# Quality of Life in Patients With a Major Mental Disorder in Singapore

**DOI:** 10.3389/fpsyt.2018.00727

**Published:** 2019-01-18

**Authors:** Carol C. Choo, Peter K. H. Chew, Cyrus S. Ho, Roger C. Ho

**Affiliations:** ^1^Department of Psychology, College of Healthcare Sciences, James Cook University, Singapore, Singapore; ^2^Department of Psychological Medicine, National University of Singapore, Singapore, Singapore; ^3^Center of Excellence in Behavioral Medicine, Nguyen Tat Thanh University, Ho Chi Minh City, Vietnam

**Keywords:** schizophrenia, depression, Quality of Life, Physical Component Summary, Mental Component Summary (MCS)

## Abstract

**Background:** There has been a paradigm shift in mental health service delivery, from a focus on reducing symptoms to a more holistic approach, which considers Quality of Life (QoL).

**Method:** This study aimed to explore prediction of Quality of Life (QoL) in Asian patients with a major mental disorder i.e., depression or schizophrenia in Singapore. In the current study, there were 43 patients (65.1% females) with depression. Their ages ranged from 18 to 65 (*M* = 44.63, *SD* = 12.22). The data were combined with the data on patients with schizophrenia, where there were 43 patients (65.1% females) with schizophrenia, their ages ranging from 18 to 65 (*M* = 44.60, *SD* = 12.19).

**Results:** The components of QoL were examined i.e., Physical Component Summary (PCS) and Mental Component Summary (MCS). For all patients, social support and age accounted for 17.3% of the variance in PCS, *F*_(2, 83)_ = 8.66, *p* < 0.001. For patients with depression, disorder severity, age, and duration of treatment accounted for 48.3% of the variance in PCS, *F*_(3, 39)_ = 12.15, *p* < 0.001. For patients with schizophrenia, education (Primary or Lower vs. Post-Secondary or Higher) and emotional coping accounted for 21.3% of the variance in PCS, *F*_(2, 40)_ = 5.40, *p* < 0.01. For all patients, self-efficacy and age accounted for 27.0% of the variance in MCS, *F*_(2, 83)_ = 15.37, *p* < 0.001. For patients with depression, disorder severity accounted for 45.6% of the variance in MCS, *F*_(1, 41)_ = 34.33, *p* < 0.001. For patients with schizophrenia, number of hospitalizations accounted for 18.5% of the variance in MCS, *F*_(1, 41)_ = 9.29, *p* < 0.01.

**Conclusion:** The findings were discussed in regards to implications in interventions to enhance QoL of patients with schizophrenia and depression in Singapore.

## Introduction

There has been a paradigm shift in mental health service delivery, from a focus on reducing symptoms to a more holistic approach, which considers Quality of Life (QoL) and overall functioning (1). More mental health services are adopting the recovery paradigm (2), focusing on rehabilitation (3), and QoL is an increasingly critical outcome of mental healthcare (4).

Lower Quality of Life (QoL) had been associated with major mental disorders, such as depression (5, 6) and schizophrenia (4, 7). Both mental disorders are of concern (8) to clinicians working in mental health services: Schizophrenia is the most common diagnosis among hospitalized psychiatric patients, and it is a chronic mental disorder with a debilitating course (9). Functional impairment is high, leading to lost wages and work impairment, with related personal, societal, and economic burdens (10, 11). Depression is prevalent (12), a leading cause of disability worldwide (13) and highlighted as a growing public health concern in both Western (14) and Asian studies (15), with depressed patients reporting lower QoL compared to other mental disorders, including schizophrenia (16). Domestic life, work, and interpersonal activities were the most affected functional domains in depression (17), with adverse outcomes in low education, marital disruption, unstable employment, risk of secondary disorders, and early mortality due to suicide (18); while interpersonal and affective problems were found consistently across countries, cross-national variations are noted in other domains. QoL is a culturally sensitive construct (8), and it should not be assumed that conclusions from Western studies could be generalized across to Asian populations. A recent study (8) examined the QoL of Taiwanese patients with chronic mental disorders, namely schizophrenia and affective disorders. Besides disease factors, a range of psychosocial factors was found to contribute to QoL. Many of these factors were culturally sensitive; recent research done in Singapore cautioned against the generalization of research done in other countries to the Asian population in Singapore (19), without due consideration of the local sociocultural context (20). Studies related to mental disorders and QoL in Asia (21) and Singapore (22) focused mainly on patients with one mental disorder, namely schizophrenia (21) and depression (22). A study done in Taiwan (8) examined patients with schizophrenia and affective disorders, but generalizability of the results to Singapore remained unexplored. The question if there might be differences in configuration of factors contributing to QoL across the major mental disorders named above, specifically schizophrenia and depression, remains largely unexplored in Singapore. This research question is relevant and useful to clinicians working with the recovery paradigm in mental health service delivery, to facilitate the management of major mental disorders (4).

Usage of a repertoire of more than one coping strategy led to greater effectiveness in managing mental disorders (23). Individuals with schizophrenia tended to use emotion-focused and avoidant coping (24), however it is unclear if these strategies are helpful or maladaptive. Maladaptive coping strategies might adversely disturb the overall functioning of people with mental disorders (25) and add to caregiver burden (10). Individuals with mental disorders tended to report lower distress if they had greater social support (26) and employ coping strategies (23). However, the specific mechanisms and interactions of these psychosocial factors in influencing the QoL of individuals with different types of mental disorders remains largely unexplored. Coping flexibility includes a broad coping repertoire, a well-balanced coping profile, cross-situational variability in strategy deployment, and a good strategy-situation fit (27). Possible cultural influences were suggested by differences in coping flexibility and psychological adjustment in samples from countries lower or higher in individualism.

Research is needed in the local population, as it cannot be assumed that research findings conducted in other countries are also relevant to the local Asian population in Singapore. In Western samples, functional support concerning quality of relationships and perceived social support were related to QoL (28, 29) but this phenomenon was not found in Asian samples (30). In contrast, conclusions that were consistent across samples included the association between self- efficacy with lowered stress and enhanced mental health and QoL in Western samples (31, 32), as well as QoL in Asian samples (33, 34). Recent local research suggested that education level contributed to QoL (35). Other recent Asian studies suggested that duration of illness (36) and social support (37) were prominent in QoL (23), together with possible influences from demographic variables such as gender (36–38) and age (2). Many factors could contribute to QoL in mental disorders, which makes comparing results across different studies difficult, especially when the methodologies, designs and measures employed were also different.

The current study would analyze the QoL of Singaporean patients with depression, and schizophrenia. This study aimed to address the current gap in local literature by examining the ability of available variables namely demographic characteristics such as age, gender, and education, and psychosocial variables namely social support, self-efficacy, and coping styles, as well as disorder severity, and number of hospitalizations in predicting QoL for patients with depression and schizophrenia. The data sets would be combined for patients with depression and schizophrenia. Similar to a recent study in Singapore (22), QoL would be broken down into Physical Component Summary (PCS) and Mental Component Summary (MCS). It was hypothesized that self-efficacy, coping (39), social support (25), and education (35) would positively predict PCS and MCS, while number of hospitalizations and disorder severity (33, 36) would negatively predict PCS and MCS. The study would analyze the prediction of PCS and MCS in patients with a major mental disorder i.e., depression and schizophrenia as a combined sample, and also separately.

## Materials and Methods

### Procedure

Ethics approval was obtained from the Domains-Specific Review Board of a large teaching hospital in Singapore. Convenience sampling was used. Forty-three patients diagnosed with depression and 43 patients diagnosed with schizophrenia were recruited from an outpatient clinic and psychiatric ward of a large teaching hospital in Singapore from January to May 2010. Eligibility criteria for the study included the following: (a) above the legal age of consent, (b) able to understand English and/or Mandarin, and (c) absence of intellectual impairment. The patients' psychiatric symptoms, subjective Quality of Life (QoL), self-efficacy, perceived social support, and coping style were investigated with the use of self-report surveys. The survey included items on: Demographic background: age, gender, and education, (2) Nature and history of presenting problems, (3) The patient's current psychiatric condition, perceived QoL, self-efficacy, social support, and coping styles.

Clinical staff conducted the recruitment. After interested individuals returned their signed consent forms, they received the survey. After completion of the survey, participants were debriefed, and thanked for their participation.

In the current study, there were 43 patients (65.1% females) with depression, their ages ranged from 18 to 65 (*M* = 44.63, *SD* = 12.22). The data would be combined with the data on patients with schizophrenia, there were 43 patients (65.1% females) with schizophrenia, their ages ranged from 18 to 65 (*M* = 44.60, *SD* = 12.19). The characteristics of the combined sample are presented in Table 1.

**Table 1:** Characteristics of the sample.

	**All**		**Depression**		**Schizophrenia**	
	***n***	**%**	***n***	**%**	***n***	**%**
**GENDER**						
Male	30	34.9	15	34.9	15	34.9
Female	56	65.1	28	65.1	28	65.1
**EDUCATION**						
Primary or Lower	15	17.4	11	25.6	4	9.3
Secondary School	41	47.7	16	37.2	25	58.1
Post-Secondary or Higher	30	34.9	16	37.2	14	32.6
**MARITAL STATUS**						
Single	40	46.5	14	32.6	26	60.5
Married	38	44.2	24	55.8	14	32.5
Divorced / Separated	8	9.3	5	11.6	3	7.0
**EMPLOYMENT**						
Employed	42	48.8	24	55.8	18	41.9
Unemployed	44	51.2	19	44.2	25	58.1
	***M***	***SD***	***M***	***SD***	***M***	***SD***
Age	44.62	12.13	44.63	12.22	44.60	12.19
**DURATION OF (IN DAYS)**						
Condition	103.91	105.30	74.47	65.09	133.35	128.18
Treatment	47.83	79.56	61.99	63.93	33.67	91.18
No. of hospitalizations (past one year)	0.36	0.57	0.16	0.43	0.56	0.63
Disorder severity	16.52	9.61	9.63	6.59	23.42	6.78

### Materials

The 4-page survey included items on demographic background and the following questionnaires. Disorder severity for depression was measured using the Depression Anxiety Stress Scale, DASS-21 (40). Items were scored on 4-point scales, whereby higher overall scores denoted poorer management of symptoms. High validity and reliability were shown in Asian samples from Singapore (22) and Malaysia (41). For individuals with schizophrenia, the psychiatric condition was assessed using the Brief Psychiatric Rating Scale, BPRS (42). The scale comprised 18 items rated on 7-point scales of severity, based on behavioral anchors. An overall measure of symptom severity was obtained by summing up the scores of all 18 items. Quality of Life (QoL) was measured using the Short Form Health Status Questionnaire (SF-12) (43). Eight domains of health which included physical functioning, role limitations due to physical and emotional problems, bodily pain, general health, vitality, social functioning, and mental health were assessed across 12 items. Appropriate items were summed to produce two factors: Physical Component Summary (PCS) and Mental Component Summary (MCS), with higher scores indicating better perceived QoL. High test/retest validity as well as good internal reliability were shown with a Chinese sample (44). Level of functional social support was assessed by a four-item measure designed to detect various archetypal functions of informational support, emotional support, positive social interaction (45), and ability to promote self-disclosure (46). This measure was scored on 5-point Likert scales, whereby higher overall scores showed perception of greater support from spouses, families, and/or friends. The internal reliability was 0.85 (22). Self-efficacy was measured using the Self-Mastery Scale SMS (47). The SMS comprised seven items scored on 5-point Likert scales, whereby higher overall scores showed greater tendency to perceive life events as under self-control rather than that of external forces. High face validity and good internal reliability were demonstrated in a Thai study (48).

## Results

The data were analyzed using SPSS Version 23 with the alpha level set at 0.05. The means and standard deviations of the variables are presented in Table 2. The categorical variables: Education and Marital Status, were re-coded with Primary or Lower and Single as the comparison group, respectively.

**Table 2:** Means and standard deviations of the variables.

	**All**		**Depression**		**Schizophrenia**	
	***M***	***SD***	***M***	***SD***	***M***	***SD***
**PREDICTORS**						
Social Support	14.27	2.96	19.00	13.95	14.58	2.74
Self-Efficacy	21.55	3.92	28.00	20.60	22.49	3.08
Active Coping	3.65	0.90	3.47	0.96	3.84	0.81
Emotional Coping	3.47	0.95	3.47	0.88	3.47	1.03
Support Coping	3.64	1.09	3.37	1.16	3.91	0.97
Avoidant Coping	2.94	1.21	3.00	1.11	2.88	1.31
**CRITERION**						
PCS	44.48	10.23	42.32	10.04	46.65	10.08
MCS	41.54	12.09	36.99	11.85	46.09	10.63

A series of six forward stepwise multiple regressions were used to assess the ability of the predictor variables (characteristics of the sample and the predictors, with a total of 17 predictor variables) to predict Physical Component Summary (PCS) and Mental Component Summary (MCS). The assumptions of multiple regression were examined. First, an inspection of the Durbin-Watson statistic showed that the independence of errors assumption is satisfied (i.e., the values are close to 2). Second, an inspection of the correlation matrix, Tolerance values, and Variance Inflation Factor (VIF) showed that the multicollinearity assumption was satisfied. Third, an inspection of the maximum Cook's Distance showed that there were no univariate outliers (i.e., the values were <1). Fourth, an inspection of the maximum Mahalanobis distance showed that there were no multivariate outliers because the values were less than the critical value of 27.59 (given *df* = 17 and alpha = 0.05). Fifth, an inspection of the Normal Probability Plot (P-P) showed that the normality assumption was satisfied. Lastly, the scatterplot was inspected to assess the homoscedasticity assumption. While the assumption was satisfied for the all patients, and patients with depression, it was violated for patients with schizophrenia. However, the data was not transformed since the assumption was satisfied for the other groups. Hence, the results from patients with schizophrenia should be interpreted with caution.

### Physical Component Summary PCS

For all patients, social support and age accounted for 17.3% of the variance in PCS, *F*_(2, 83)_ = 8.66, *p* < 0.001. For patients with depression, disorder severity, age, and duration of treatment accounted for 48.3% of the variance in PCS, *F*_(3, 39)_ = 12.15, *p* < 0.001. For patients with schizophrenia, education (Primary or Lower vs. Post-Secondary or Higher) and emotional coping accounted for 21.3% of the variance in PCS, *F*_(2, 40)_ = 5.40, *p* < 0.01. The results are presented in Table 3.

**Table 3:** Stepwise multiple regression with PCS and MCS as the dependent variables.

	**Beta**	***p***
**DEPENDENT VARIABLE: PCS**		
**All Patients**		
Social Support	0.30	0.004
Age	−0.27	0.008
**Patients with Depression**		
Disorder severity	−0.65	0.000
Age	−0.42	0.002
Duration of treatment	−0.27	0.034
**Patients with Schizophrenia**		
Education (Primary or Lower vs. Post-Secondary or Higher)	−0.35	0.018
Emotional Coping	0.33	0.025
**DEPENDENT VARIABLE: MCS**		
**All Patients**		
Self-Efficacy	0.46	0.000
Age	0.26	0.008
**Patients with Depression**		
Disorder severity	−0.68	0.000
**Patients with Schizophrenia**		
No. of hospitalizations (past 1 year)	−0.43	0.004

### Mental Component Summary MCS

For all patients, self-efficacy and age accounted for 27.0% of the variance in MCS, *F*_(2, 83)_ = 15.37, *p* < 0.001. For patients with depression, disorder severity accounted for 45.6% of the variance in MCS, *F*_(1, 41)_ = 34.33, *p* < 0.001. For patients with schizophrenia, number of hospitalizations accounted for 18.5% of the variance in MCS, *F*_(1, 41)_ = 9.29, *p* < 0.01. The results are presented in Table 3.

## Discussion

This study aimed to explore prediction of Quality of Life (QoL) in 86 patients with schizophrenia and depression in Singapore. As hypothesized, the models were significant in predicting Physical Component Summary (PCS) and Mental Component Summary (MCS). For all patients, social support positively and age negatively predicted PCS. For patients with depression, disorder severity, age, and duration of treatment negatively predicted PCS. For patients with schizophrenia, education (Primary or Lower vs. Post-Secondary or Higher) and emotional coping predicted PCS. For all patients, self-efficacy and age positively predicted MCS. Self-efficacy and age positively predicted MCS for all patients. Disorder severity negatively predicted MCS for patients with depression. For patients with schizophrenia, number of hospitalizations negatively predicted MCS.

The results suggested psychosocial variables, namely social support and self-efficacy, need to be considered in their contribution to QoL for patients with depression and schizophrenia. The findings are consistent with previous literature associating psychosocial variables with QoL (35, 39). As compared with results from our study on QoL of patients with schizophrenia, prediction of QoL (i.e., MCS and PCS) differed for patients with depression and schizophrenia. For patients with depression, disorder severity, age, and duration of treatment negatively predicted PCS; while disorder severity negatively predicted MCS. For patients with schizophrenia, education and emotional coping predicted PCS, while number of hospitalizations negatively predicted MCS. This is consistent with previous research (22, 24, 26).

For patients with depression, disorder severity and duration of treatment might be associated with the chronicity of the mental disorder or it could be indicative of treatment resistance, conditions that are harder to treat e.g., dysthymia or other co-morbid conditions (16) or symptoms not investigated in the current study, and detailed below. QoL could be affected by relapses caused by residual mood symptoms and insomnia, which were not explored in this study (48). Somatic symptoms were also frequent among Asian patients with depression, were associated with greater clinical severity, and lower response and remission rates (49), pain symptoms also impacted on severity of depression and QoL (50). Other psychosocial variables that might affect impact of depression include income status (38), financial and marital problems, self-esteem (14), and religiosity (51) previously investigated but not explored here.

Future studies could employ in-depth interviews to elicit detailed information, to identify different layers of factors contributing to QoL (i.e., MCS and PCS) of patients with schizophrenia and depression. Such factors to consider might include the social cultural environment, and other contextual factors that might impact on patients, e.g., socioeconomic status, ethnicity (52), and stigma, in addition to detailed features of the mental disorder, co-morbid conditions or somatic, and residual symptoms contributing to relapse.

The current findings have implications for psychosocial interventions and mental health service delivery to patients with schizophrenia and depression in Singapore, and offer preliminary evidence to support the important role that allied health professionals play in the delivery of psychosocial interventions targeted toward enhancing social support and self-efficacy. In alignment with recent paradigm changes in mental health service delivery (1), clinicians could take on a case management role, working closely with community agencies and allied health professional to provide holistic care for those with mental disorders. Such holistic interventions could include peer led groups, or social groups, where individuals with mental disorders could be encouraged to engage in leisure activities typically used for emotion-focused coping or avoidance coping. In alignment with the contemporary focus on recovery (2) and rehabilitation (3), mental health promotion could be targeted toward reducing stigma related to mental disorders, and family support could be encouraged by offering psychoeducation to family members of individuals with mental disorders, on how best they could support these individuals in the community. These recommendations are consistent with suggestions made in other recent Asian studies to promote family interventions (53) and to reduce stigma (4).

The current findings added to QoL research, providing more insight for clinicians and allied health professionals to understand how they might work in a more targeted way, with local patients with debilitating mental disorders, specifically schizophrenia and depression to enhance QoL. Patients with depression might benefit from evidence-based therapies e.g., Cognitive Behavioral Therapy, shown to be efficacious in reduction of core mood symptoms and residual symptoms, together with empowering patients with strategies for relapse prevention (54). Patients with schizophrenia and their families might benefit from psychoeducation on relapse prevention to reduce number of hospitalizations.

Limitations to the study included the following. A lack of a control group limited how the data could be interpreted. Future research could compare data collected from patients with schizophrenia or depression with matched healthy controls on key variables, for better interpretation of data. The current study used convenience sampling on two major mental disorders, however, these mental disorders are very different, with different treatments and outcomes. Future research could examine mental disorders in the same spectrum, e.g., schizophrenia spectrum and other psychotic disorders; or the spectrum of depressive disorders. Information was not available regarding the response rate, or the prescribed treatment, future research could endeavor to collect more comprehensive information, for better interpretation of the results. It is difficult to ascertain causality in cross-sectional research. Whilst our study used statistical analysis to predict MCS and PCS, a longitudinal methodology could enhance our interpretation of the contribution of key variables across time. In addition, other contextual factors might impact on patients, e.g., socioeconomic status, ethnicity (52), and stigma. However, such contextual information was not collected in this study, future research could employ in-depth interviews to elicit the intricate interplay among contextual factors, which could be confounding variables, and might limit the generalizability of our results. Other limitations of the study included the reliance on self-report, and sampling from one hospital. With the patients' consent, family members or clinical staff could be interviewed for corroboratory information. The data was collected in 2010 in one hospital, this data was the only data set with the key variable of interest, made available to the researchers, the data with the key variables was not available after the stipulated period. Future research could replicate the study with a more recent sample and a larger sample size of patients from polyclinics and both private and public hospitals in Singapore or consider combining data with similar research in other Asian countries.

## Conclusion

The current findings added existing to QoL research and shed additional insight for clinicians to understand how they might work in a more targeted way with local patients with debilitating mental disorders, namely schizophrenia and depression, in order to enhance Quality of Life (QoL). The findings also offer preliminary evidence to support the important role that allied health professionals could play in the delivery of psychosocial interventions targeted toward enhancing social support and self-efficacy.

## Author Contributions

CC, RH, CH, and PC conceptualized the paper, participated in revisions, and approved the final manuscript. CH and RH designed the study and collected data. PC analyzed data. RH gave access to the data. CC compiled data, wrote the manuscript, and applied for funding.

### Conflict of Interest Statement

The authors declare that the research was conducted in the absence of any commercial or financial relationships that could be construed as a potential conflict of interest.
